# Genome-wide association mapping of genomic regions associated with drought stress tolerance at seedling and reproductive stages in bread wheat

**DOI:** 10.3389/fpls.2023.1166439

**Published:** 2023-05-12

**Authors:** S Srinatha Reddy, Dinesh Kumar Saini, G Mahendra Singh, Sandeep Sharma, Vinod Kumar Mishra, Arun Kumar Joshi

**Affiliations:** ^1^ Department of Genetics and Plant Breeding, Institute of Agricultural Sciences, Banaras Hindu University, Varanasi, India; ^2^ Department of Plant Breeding and Genetics, Punjab Agricultural University, Ludhiana, Punjab, India; ^3^ Borlaug Institute of South Asia (BISA), NASC Complex, DPS Marg, New Delhi, India; ^4^ CIMMYT, NASC Complex, DPS Marg, New Delhi, India

**Keywords:** drought, hydroponics, seedling, reproductive, wheat, GWAS, haplotype

## Abstract

Understanding the genetic architecture of drought stress tolerance in bread wheat at seedling and reproductive stages is crucial for developing drought-tolerant varieties. In the present study, 192 diverse wheat genotypes, a subset from the Wheat Associated Mapping Initiative (WAMI) panel, were evaluated at the seedling stage in a hydroponics system for chlorophyll content (CL), shoot length (SLT), shoot weight (SWT), root length (RLT), and root weight (RWT) under both drought and optimum conditions. Following that, a genome-wide association study (GWAS) was carried out using the phenotypic data recorded during the hydroponics experiment as well as data available from previously conducted multi-location field trials under optimal and drought stress conditions. The panel had previously been genotyped using the Infinium iSelect 90K SNP array with 26,814 polymorphic markers. Using single as well as multi-locus models, GWAS identified 94 significant marker-trait associations (MTAs) or SNPs associated with traits recorded at the seedling stage and 451 for traits recorded at the reproductive stage. The significant SNPs included several novel, significant, and promising MTAs for different traits. The average LD decay distance for the whole genome was approximately 0.48 Mbp, ranging from 0.07 Mbp (chromosome 6D) to 4.14 Mbp (chromosome 2A). Furthermore, several promising SNPs revealed significant differences among haplotypes for traits such as RLT, RWT, SLT, SWT, and GY under drought stress. Functional annotation and *in silico* expression analysis revealed important putative candidate genes underlying the identified stable genomic regions such as protein kinases, O-methyltransferases, GroES-like superfamily proteins, NAD-dependent dehydratases, etc. The findings of the present study may be useful for improving yield potential, and stability under drought stress conditions.

## Introduction

Drought stress is defined as a lack of water that causes dramatic morphological, physiological, biochemical, and molecular changes in plants (Sallam et al., 2019) that restrict plant growth, development, and production. The global warming and climate change are expected to increase the frequency of droughts, resulting in reduced crop production (Sallam et al., 2019). Wheat (*Triticum aestivum* L.) is an important staple food crop that supplies more protein (approximately 23%) than all animal sources and accounts for more than 20% of human calorific intake (http://www.fao.org/faostat/en/#data/QC; Pal et al., 2022). In the past 50 years, global wheat production increased from 343 million tonnes in 1972 to 770 million tonnes in 2021 (https://knoema.com/atlas/World/topics/Agriculture/Crops-Production-Quantity-tonnes/Wheat-production). During the previous century, there was annual progress of 0.3% to 1.0% in the genetic improvement of wheat’s grain yield (Graybosch and Peterson, 2010; Pal et al., 2022). Nevertheless, it has decreased recently, largely as a result of the limited genetic diversity in the breeding populations, climate change associated abiotic and biotic stresses (Tanin et al., 2022), and lack of novel breeding techniques in many programs. Notably, with the unusual constraints posed by climate change, there is a need to increase wheat yield to feed the growing human population.

Wheat crop is sensitive to heat and drought stresses (Li et al., 2011), particularly during the flowering and grain development stages and both yield and grain quality gets impacted (Sallam et al., 2018). According to an estimate, drought and heat stress accounted for 40% of annual production variability in major wheat producing countries (Zampieri et al., 2017). Several studies have demonstrated drought to be a potential threat to wheat production in a populous country like India (Joshi et al., 2007), South Asia and other similar global ecologies (Singh et al., 2007). Wheat demand is expected to increase by 60% by 2050, but production may decrease by 29% due to climate change-induced environmental stresses (Manickavelu et al., 2012; Kulkarni et al., 2017). These forecasts suggest that understanding wheat adaptation to drought and developing drought-tolerant varieties is crucial for maintaining and increasing global wheat production.

Under drought stress, wheat plants have a restricted ability to absorb nutrients and have low photosynthetic efficiency (Praba et al., 2009). It can also decrease organ size (leaf, spikes, etc.) and growth period of various developmental stages (tillering, booting, heading, anthesis, grain filling, maturity, etc.) (Hossain and Teixeira, 2013; Ihsan et al., 2016). Sensitivity of wheat to drought stress results in interrupted metabolic processes and a shorter life cycle which ultimately reduces plant biomass and grain yield (Itam et al., 2020). Drought tolerance is a complex quantitative trait governed by many genes, some of which have minor effects while others have major effects (Kumar et al., 2012). Further, it has low heritability owing to its polygenic inheritance and significant genotype by environment interaction (Sukumaran et al., 2018; El Gataa et al., 2022; Rabieyan et al., 2022). Drought tolerance can be improved genetically by identifying sources of stress-tolerant traits and/or genotypes, and introgressing and mobilizing the genomic regions controlling these traits into locally adapted cultivars (Shelake et al., 2022). The difficulty in utilizing this approach in breeding programs is the absence of proper knowledge about the most relevant traits for different stress scenarios and their detection in a quick, efficient and cost-effective manner. Our knowledge of the genetic underpinnings and molecular basis of complex traits like drought tolerance has increased as a result of recent developments in high-throughput genotyping (Saini et al., 2022) and phenotyping techniques (Gill T. et al., 2022; Xiao et al., 2022).

Bi-parental mapping is an efficient method for dissecting the genetic architecture of different complex traits in wheat (Singh et al., 2022; http://www.wheatqtldb.net/). However, it generally produces low-resolution maps and mostly uses few polymorphic markers with less genome coverage, which results in a non-significant association of the identified markers with the target traits, creating ambiguity in selecting for marker assisted selection programs. As a result, current genomic mapping attempts are shifting from conventional bi-parental mapping to linkage disequilibrium (LD) based mapping including GWAS which offers two unique advantages- (i) it eliminates the costs and time associated with population development, and (ii) it effectively utilizes the numerous historical crossover events that occurred in the diverse association panel used, and as a result, provides high mapping resolution (Saini et al., 2022; Xiao et al., 2022).

Several earlier GWAS studies have detected the genomic regions associated with traits contributing to drought stress tolerance in wheat either at seedling (e.g., Maulana et al., 2020; Danakumara et al., 2021; Sallam et al., 2022) or at reproductive stage (e.g., Gataa et al., 2021; El Gataa et al., 2022), however, only a few studies identified genomic regions for traits recorded on both seedlings and reproductive stages (e.g., Rufo et al., 2020). The establishment of seedlings is believed to have significant effects on crop stand and therefore crop yield. It has been suggested that early vegetative stages, such as the seedling stage of wheat, have greater stress avoidance and resilience (Sallam et al., 2018). Early generation screening and selection using hydroponics was found successful because it is feasible and practical to perform mass phenotyping and high-throughput characterization of shoot and root related traits during the seedling stage (Ayalew et al., 2015; Islam et al., 2015). These findings necessitated further research into the relationship between drought tolerance performances at various stages of wheat development. Further, as mentioned above, there is a paucity of information on the common genetic determinants conferring tolerance to drought at both seedlings and reproductive stages. Given this, the current study aimed to identify and compare significant genomic regions that were responsible for drought tolerance at the seedling and adult plant stages, which could be used in wheat breeding to increase resilience to drought stress.

The objective of the study was to identify the significant marker-trait associations (MTAs) for drought tolerance at seedling (in hydroponics: PEG 8000 induced drought) and at the reproductive stage (in field conditions) and to find important haplotypes associated with drought stress tolerance at the both developmental stages. GWAS was performed for different seedling traits, including chlorophyll content (CL), shoot length (SLT), root length (RLT), shoot weight (SWT) and root weight (RWT) and several adult plant traits, such as days to heading (DTH), days to maturity (DTM), plant height (PHT), thousand kernel weight (TKW), grain yield (GY), normalized difference vegetative indices at heading (NDVIH), grain filling (NDVIGF), and maturity (NDVIM) stages, and canopy temperature depression at heading (CTDH), grain filling (CTDGF) and maturity (CTDM) stages recorded under both optimum and drought stress conditions.

## Materials and methods

### Plant material

We evaluated 294 diverse wheat genotypes, a subset from the Wheat Associated Mapping Initiative (WAMI) Panel under field conditions. This WAMI panel was obtained from CIMMYT international nurseries, which are distributed globally each year (Lopes et al., 2015). The genotypes with similar heading dates (within one week) were screened and a subset of 192 genotypes was chosen for the subsequent experiments. The experiment was carried out under hydroponics conditions to assess the responses of genotypes to drought stress at seedling stage.

### The layout of the experiments and drought treatment

The genotypes were evaluated in a controlled hydroponic system at the Department of Genetics and Plant Breeding, Institute of Agricultural Sciences, Banaras Hindu University, Varanasi, during the year 2020-21. The plants were grown in half-strength Hoagland solution and the experiment was set up in a completely randomized design (Hoagland and Arnon, 1950) (DC and DI, 1950) (Hoagland and Arnon, 1950). Hoagland solution is a hydroponic nutrient solution and one of the best nutrient compounds for plant growth. Hoagland solution contains all of the nutrients required for plant growth and is appropriate for the growth of many different plant species. Seeds were initially sterilized with sodium hypo chloride solution (1% for 5 min) and rinsed with distilled water for times. The overnight soaked seeds were placed in a petri plate lined with Whatman filter paper. Petri plates were watered for three consecutive days to promote seed germination. The germinated seeds were then put in sterile polystyrene tubes fitted with thermacol and holes, floated on three litres of half strength Hoagland solution, and placed in rectangular plastic trays with a four-litre capacity. The light intensity of 250µmolm^-2^s^-1^ with 10/14hrs dark and light duration was maintained using fluorescent tubes (Ayalew et al., 2015). Drought treatment was imposed on 14 days old seedlings by dissolving 20% PEG-8000 (Poly Ethylene Glycol) in Hoagland solution. The treatment was extended for seven days and then replaced with only Hoagland solution, with readings taken after three days of stress recovery. While under control conditions, seedlings were grown by replacing the growing solution with only fresh Hoagland solution every week for 24 days. The data was recorded on CL (using SPAD-502Plus, Konica Minolta, Inc. made in Japan), SLT, RLT, SWT and RWT under both control and stress conditions.

The data available on different morpho-physiological traits recorded during field trials conducted at three locations in India (Varanasi, Pune, and Jabalpur) during the cropping season 2021-22 was also considered for the current study. These morpho-physiological traits included the following: DTH, DTM, PHT, TKW, GY, NDVIH, NDVIGF, NDVIM, CTDH, CTDGF and CTDM (the details on observations recorded and the phenotypic data for the field experiments are provided in one of our earlier study (Reddy et al., 2023).

### Statistical analysis

Following the testing of the data for normal distribution, the analysis of variance (ANOVA) was performed. All descriptive statistics were calculated using Microsoft Excel 2016, and ANOVAs were performed using Genstat 18^th^ edition (32 bit). Further, stress indices were estimated for all the traits at the seedling stage. Stress indices and correlation was calculated using OriginPro 2022 (https://www.originlab.com/). Details on different stress indices calculated are provided in **Table 1**.

**Table 1:** Details on different stress indices.

S. No.	Stress indices	Formula	Reference
1.	Drought resistance index (DI)	(Ys×(Ys/Yp)/ $\bar{Y}$ s	(Lan, 1998)
2.	Geometric mean productivity (GMP)	√(Ys × Yp)	(Fernandez, 1992)
3.	Mean productivity index (MPI)	(Ys + Yp)/2	(Rosielle and Hamblin, 1981)
4.	Relative drought index (RDI)	(Ys/Yp)/( $\bar{Y}$ s/ $\bar{Y}$ p)	(Fischer and Maurer, 1978)
5.	Stress tolerance index (STI)	(Ys×Yp)/( $\bar{Y}$ p)ˆ2	(Fernandez, 1992)
6.	Stress index (SI)	Ys/Yp	(Bouslama and Schapaugh, 1984)

Where Ys and $\bar{Y}$ s is the trait value and mean of the trait under drought treatment, respectively and Yp and $\bar{Y}$ p is the value for the same trait and mean value under the control condition, respectively.

### SNP genotyping and population structure

The WAMI panel was previously genotyped using a high-density illumina 90K infinium SNP array which led to the identification of a total of 26,814 polymorphic SNPs. The missing data points in the genotypic data file were imputed using LD-KNN imputation algorithm available in TASSEL. Only 20,713 SNPs were considered for the analysis out of the 26,814 SNPs that had physical positions available from the Wheat URGI database (https://wheat-urgi.versailles.inra.fr/) based on IWGSC ref seq v1.1.

Principal component analysis (PCA) of the imputed genotypic data was then performed in R to examine population structure using the Genomic Association and Prediction Integrated Tool (GAPIT) version 3.0 (Wang and Zhang, 2021). Population structure was also analyzed using STRUCTURE version 2.3.4, a bayesian model-based clustering program that assumes an admixture model (Pritchard et al., 2000) and utilized ten subgroups (K = 1-10), each with five independent runs, with a burn-in period of 10,000 iterations followed by 10,000 Monte-Carlo iterations. To infer the most likely number of subpopulations, an *ad hoc* statistic (DeltaK) was used, which utilized the rate of alterations in the log probability between runs utilizing successive K-values (Evanno et al., 2005) with STRUCTURE HARVESTER (Earl and Bridgett, 2012). Then, GWAS analysis was performed using the 20,713 high-quality physically anchored SNPs and BLUE (best linear unbiased estimates) values estimated using the data available from three individual environments (Varanasi, Jabalpur, and Pune) for all the traits (recorded under both optimum and drought stress conditions). The BLUE values were computed utilizing META-R which uses LME4 R-package for linear mixed model analysis (Bates et al., 2015).

### Linkage disequilibrium (LD) analysis

LD analysis was performed using TASSEL v5.0 software for the whole genome and individual chromosomes by estimating r^2^ values for all pairwise marker comparisons with a sliding window size of 50 SNPs. The LD decay over genetic distance was calculated by fitting a nonlinear model with the modified Hill and Weir approach (Hill and Weir, 1988), with the r^2^ threshold set at 0.2 and r^2^ equalling half the decay distance. Using R, the LD decay distance for each chromosome and the whole genome was shown (R Core Team, 2013).

### Genome-wide association mapping, assessment of the effects of different alleles of MTAs on traits and haplotype analysis

For GWAS analysis of each trait, two methods were used: Single locus mixed linear model and multi-locus mixed model. The mixed linear model (MLM) was used in the single-locus method to account for population stratification (K-PC model) (Yu et al., 2006), whereas the multi-locus mixed model employed the Bayesian-information and Linkage-disequilibrium Iteratively Nested Keyway (BLINK) and Fixed and random model Circulating Probability Unification (FarmCPU) methods.

All three models (viz., MLM, BLINK, and FarmCPU) were implemented in the R environment using the GAPIT version 3.0 (with default parameters) and involved the first two PCs to compensate for population structure, as determined by evaluation of the scree plot and the DeltaK statistic obtained from STRUCTURE analysis (Wang and Zhang, 2021). The Bonferroni-corrected threshold of P<0.1 was calculated as -log10(P) = 5.61, but it turns out that this threshold is too strict because it takes into account all SNPs in the dataset instead of independent tests. Therefore, for the present study, we employed an exploratory threshold [-log10(P) = 3.00] based on independent tests to consider any SNP as significant in individual environments, as reported in some previous studies (Halder et al., 2019; Pang et al., 2020). Nonetheless, only those MTAs that exceeded this threshold and were detected for the same trait in at least two locations were reported as stable MTAs and those controlling at least two different traits were termed as pleiotropic MTAs.

Alleles of some significant SNPs identified post-GWAS analysis were utilized to evaluate their effects on the traits of interest. For each selected stable MTA, trait values for two groups of alleles (favourable versus unfavourable) were analyzed and visualized using a R package ‘ggstatsplot’ (Patil, 2021) which utilizes its in built parameters to test the statistical significance of the effect sizes. In addition, some significant and stable SNPs for selected traits were utilized for haplotype analysis. Haploview version 4.2. software (Barrett et al., 2005) was used to generate and display the LD-based haplotypes for a few selected regions. The wheat genotypes from the WAMI panel were grouped based on detected haplotypes, and trait data for each haplotype was subjected to analysis using the same R package ‘ggstatsplot’ (Patil, 2021).

### Identification of candidate genes and expression analysis

The gene models available in the overlapping region and/or within 1 Mb upstream and downstream of some of the selected stable MTAs were extracted using BioMart tool of EnsemblPlants database (http://plants.ensembl.org/index.html; Bolser et al., 2017). Based on the domains in the corresponding protein sequences, which were obtained using the InterPro database (https://www.ebi.ac.uk/interpro/; Hunter et al., 2009), functional annotations of the genes were performed.

*In silico* gene expression analysis was performed utilizing expVIP-powered wheat expression browser (http://www.wheat-expression.com; Ramírez-González et al., 2018). For this purpose, the appropriate expression datasets containing expression data related to drought stress were utilized (Liu et al., 2015; http://www.wheat-expression.com). The first RNA-seq dataset consisted of data on differential gene expression in a drought and heat-tolerant wheat cv. TAM107 was grown under drought and drought + heat stress conditions, with leaf samples collected separately at 1 and 6 hours after treatments (Liu et al., 2015). Expression data from the plants grown in normal growth conditions were used as the control (Liu et al., 2015). Another RNA-seq dataset (available at http://www.wheat-expression.com) consisted of differential expression of genes available from the plants treated with PEG, with leaf samples collected separately at 2 and 12 h after treatment.

The IDs of different genes available from the significant genomic regions associated with the target traits were uploaded as input to the expVIP-powered wheat expression browser and relevant datasets (as discussed above) were chosen for the expression analysis. Following analysis, expression data for each gene recorded under stress and control conditions [available as TPM (transcripts per million) values] were downloaded and further used to calculate the fold change in gene expression by dividing the TPM values obtained under drought stress by the TPM values obtained under control conditions. Only genes with a fold change (FC ≥ 2 or FC ≤− 2) compared to the control were considered differentially expressed. ClustVis (https://biit.cs.ut.ee/clustvis/; Metsalu and Vilo, 2015) was used to create heat maps of the expression patterns of the genes. The role of the identified genes in the regulation of different drought stress responsive traits was also ascertained using the published literature.

## Results

### Phenotypic evaluation

Analysis of variance (ANOVA) reported highly significant differences among the wheat genotypes for all the studied seedling traits under drought stress (**Table 2**). The percent decline under drought was maximum for SW (54.7%) followed by RW (46.7%), RL (45.6%), SL (33.3%) and CHL (20.4%) (**Table 3** and **Supplementary Figure 1**). Heritability values for different traits ranged from 0.5 (CL(C)) to 0.73(RLT(C)) under hydroponics (**Table 3**).

**Table 2:** ANOVA for the seedling traits recorded under water stress conditions in hydroponics experiment.

Sources of variation	Df	MS_CL	MS_SLT	MS_SWT	MS_RLT	MS_RWT
**Genotype**	191	32.249^**^	6.272^**^	0.035^**^	11.787^**^	0.009^**^
**Error**	192	5.833^**^	1.303^**^	0.006^**^	1.975^**^	0.002^**^
**Total**	383					

CL chlorophyll length, SLT shoot length, SWT shoot weight, RLT root length, RWT root weight. **P ≤ 0.01.

**Table 3:** Descriptive statistics and broad-sense heritability estimates for seedling traits (hydroponic experiment).

Trait	Unit	Mean	Min.	Max.	CD@95%	SD	CV	Heritability	%PD
CL(C)	SPAD units	36.02	24.73	46.55	3.91	2.79	7.74	0.5	21.1
CL(S)	SPAD units	28.41	17	39.5	4.85	4.37	15.38	0.69	
SLT(C)	cm	16.24	11.5	22.75	2.12	1.66	10.22	0.58	32.8
SLT(S)	cm	10.92	6.13	17	2.29	1.94	17.76	0.64	
RLT(C)	cm	11.3	4.75	23.2	4.56	4.45	39.38	0.73	46.7
RLT(S)	cm	6.02	2	15	2.77	2.62	43.52	0.72	
SWT(C)	g	0.86	0.14	2.64	0.41	0.34	39.53	0.66	62.8
SWT(S)	g	0.32	0.06	0.88	0.15	0.14	43.75	0.7	
RWT(C)	g	0.32	0.03	0.99	0.16	0.15	46.87	0.71	50
RWT(S)	g	0.16	0.02	0.43	0.09	0.08	50	0.63	

CL, chlorophyll content; SLT, shoot length; RLT, root length; SWT, shoot weight; RWT, root weight; (C) under control and (S) under drought.

### Correlation study of stress tolerance index with different seedling traits

**Figure 1:**
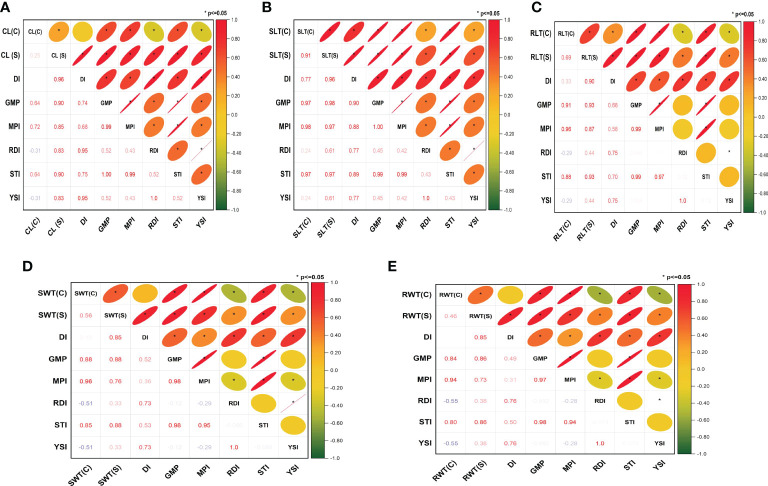
Correlation between stress indices for different traits phenotyped under hydroponics conditions: **(A)** chlorophyll SPAD readings (CL), **(B)** shoot length (SLT), **(C)** root length (RLT), **(D)** shoot weight (SWT) and **(E)** root weight (RWT). C, control; S, stress; DI, drought tolerance index; GMP, geometric mean productivity; MPI, mean productivity index; RDI, relative drought index; STI, stress tolerance index; and YSI, yield stability index. *P ≤ 0.05.

Different patterns of correlation were observed between the traits (recorded under drought stress and control conditions) and stress indices. For instance, chlorophyll SPAD readings(CL) recorded under water stress showed positive and significant correlations with all stress indices, including DI (r^2 = ^0.96), GMP (r^2 = ^0.90), MPI (r^2 = ^0.85), RDI (r^2 = ^0.83), STI (r^2 = ^0.90), and YSI (r^2 = ^0.83), whereas it showed positive correlations with some indices, including DI (r^2 = ^0.02), GMP (r^2 = ^0.64), MPI (r^2 = ^0.72), and STI (r^2 = ^0.64), and negative correlations with others such as RDI (r^2^=-0.31), YSI (r^2^=-0.31)] under control conditions (**Figures 1A–E**). Furthermore, these stress indices having a high significant correlation for all the studied traits under water stress aided in the categorization of genotypes as drought-tolerant or susceptible. The details on drought-tolerant and susceptible genotypes are presented in the **Supplementary Table 1**.

### Comparison of genotypes classified based on stress tolerance indices at seedling and adult plant stage

Under both hydroponic and field (BHU, Varanasi) conditions, six genotypes were found to be drought-tolerant, while three were reported to be drought susceptible. In contrast, five genotypes were found to be tolerant under hydroponic conditions but susceptible in the field, while six genotypes were susceptible under hydroponic conditions but tolerant in the field (BHU, Varanasi) (**Supplementary Figure 2A**). Similarly, eight genotypes were found to be drought-tolerant, while two appeared drought susceptible under both hydroponic and field conditions (ARI, Pune). Six genotypes were found to be tolerant under hydroponic conditions but susceptible under ARI, Pune field conditions. Likewise, five were susceptible under hydroponic conditions but tolerant under field conditions (ARI, Pune) (**Supplementary Figure 2B**). Under both hydroponic and field conditions, seven genotypes were found to be tolerant, while three genotypes were susceptible (BISA, Jabalpur). Three genotypes were found to be tolerant under hydroponic conditions but susceptible in the BISA, Jabalpur field, while seven genotypes were susceptible under hydroponic conditions but tolerant in the field (BISA, Jabalpur) (**Supplementary Figure 2C**).

Interestingly, four genotypes were found to be tolerant in hydroponic as well as all three field experiments. However, no genotype demonstrated consistent susceptibility to drought in hydroponic and the three field experiments. In contrast, two genotypes were found to be tolerant under hydroponic conditions but susceptible under all three field conditions, and four genotypes were susceptible under hydroponic conditions but tolerant under all three field conditions (**Supplementary Figure 2D**).

### Genotypic analysis, linkage disequilibrium (LD) and population structure

**Figure 2:**
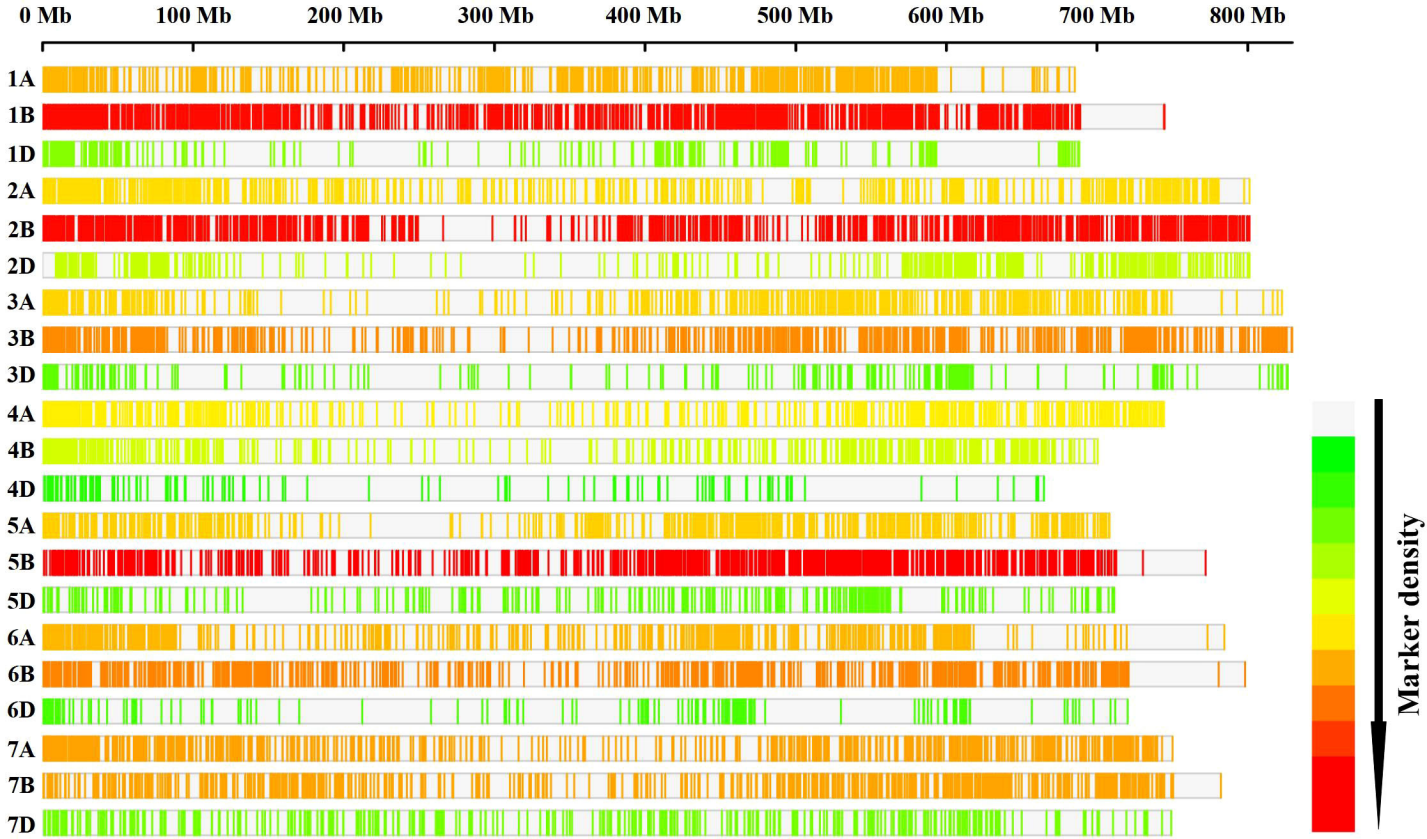
Distribution of the SNPs on different wheat chromosomes. Low-to-high SNP densities are represented by green to red.

**Figure 3:**
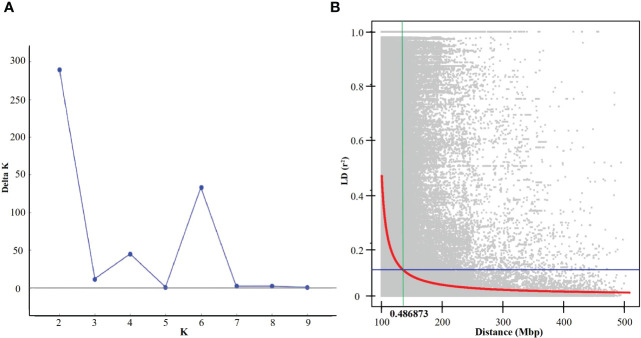
**(A)** Evanno plot of Delta-K statistic revealing the number of sub-populations in the panel, **(B)** Linkage disequilibrium (LD) in the panel for the whole genome.

Among 20,713 high-quality physically anchored SNPs, the A and B sub-genomes had six times the number of SNPs as the D sub-genome, with the B sub-genome having the most (10,120; 48.85%) and the D sub-genome having the fewest (2,736; 13.2%). The highest number of SNPs (1,841) were found on chromosome 5B, and the lowest number were found on chromosome 4D (149 SNPs) (**Figure 2**). Principal component analysis revealed significant genotype admixture, with the first two principal components accounting for most of the variance. The DeltaK statistic from the STRUCTURE analysis revealed a single peak at K = 2, indicating the presence of two subgroups in the panel (**Figure 3A**). The LD decay distance for individual chromosomes varied from 0.07 Mb (6D) to 4.14 Mb (2A). Whereas, for the whole genome, the average LD decay distance was around 0.48 Mbp (**Figure 3B**).

### Marker trait associations identified under hydroponic condition

GWAS using phenotypic data from seedlings grown in a hydroponic condition identified 95 MTAs (with MLM; including 57 MTAs for traits measured under control condition and 38 for traits recorded under stress condition), 212 (with FarmCPU; including 108 for traits measured under control condition and 104 for traits recorded under stress condition), and 212 (with BLINK; including 108 for traits measured under control condition and 104 for traits recorded under stress condition) based on the exploratory threshold of -log10(P) = 3.0 (**Supplementary Figures 3A–D**, **Supplementary Table 2**). Among these, as many as 94 MTAs were identified using all the three models (**Supplementary Table 2**). These MTAs involved 55 MTAs (located on chromosomes 1A, 3A, 3B, 4A, 4B, 5B, 5D, 7A, 7B, 7D) identified under control condition which included the following- 4 MTA for RLT, 8 for CL, 9 for RWT, 16 for SWT, and 18 for SLT and 38 MTAs (located on chromosomes 2A, 2B, 3A, 3B, 4A, 4B, 5A, 5B, 5D, 7A) identified under stress condition which included the following- 1 MTA for CL, 2 for SLT, 8 for RLT, 12 for RWT, and 15 for SWT traits (**Table 4**).

**Table 4:** Details on significant MTAs associated with seedling traits identified in hydroponics experiment conducted under water deficit conditions (detected with all three models).

Trait	Significant SNP	Chromosome	Position	-log10 (p)	SNP hits
CL (S)	Tdurum_contig49841_618	5B	38166746	3.80-4.96	exon
RLT (S)	Excalibur_c12980_2621	2A	7545148	3.08-3.27	exon
RLT (S)	RAC875_c57889_55	2A	10587192	3.38-3.62	exon
RLT (S)	Excalibur_c21051_515	3A	624831881	3.50-3.66	exon
RLT (S)	BS00021943_51	3B	69352897	3.87-4.03	exon
RLT (S)	BS00022072_51	3B	69359638	3.29-3.37	exon
RLT (S)	Ku_c61_917	4A	734000492	3.33-3.60	exon
RLT (S)	RAC875_c1817_950	7A	316021164	3.45-3.67	exon
RLT (S)	BobWhite_c1215_240	7A	664471856	3.02-3.08	exon
RWT (S)	wsnp_Ex_c34303_42642389	2B	777514133	3.49-3.94	exon
RWT (S)	Excalibur_c18318_701	4B	12528874	3.04-3.39	exon
RWT (S)	BobWhite_c162_145	4B	13977166	3.37-3.78	exon
RWT (S)	Excalibur_c8030_2139	5A	567526679	4.62-3.36	exon
RWT (S)	Tdurum_contig60421_74	5A	567526989	4.40-5.07	exon
RWT (S)	RAC875_rep_c107228_92	5A	567691316	4.42-5.10	exon
RWT (S)	Kukri_c38748_174	5A	569452729	3.27-3.67	exon
RWT (S)	BS00109396_51	5A	569456404	3.26-3.67	exon
RWT (S)	Jagger_c8122_139	5A	569463780	3.47-3.91	exon
RWT (S)	BS00022644_51	5A	569464087	3.32-3.72	exon
RWT (S)	BS00002799_51	5A	569469674	3.41-3.92	exon
RWT (S)	Kukri_c96249_58	5D	569285729	3.32-3.73	exon
SLT (S)	wsnp_Ex_c12812_20324622	4A	605731006	3.13-3.37	exon
SLT (S)	Kukri_c12563_52	4A	605732894	3.87-4.23	exon
SWT (S)	Excalibur_c42248_663	2B	6715740	3.09-3.28	exon
SWT (S)	Excalibur_c1140_1044	2B	584250260	3.38-3.63	exon
SWT (S)	IAAV3067	2B	699173434	3.06-3.27	exon
SWT (S)	wsnp_Ex_c942_1806632	2B	700207481	3.47-3.73	exon
SWT (S)	RAC875_c10132_462	2B	700208672	3.13-3.36	exon
SWT (S)	wsnp_Ex_c9025_15039930	2B	700208672	3.48-3.73	exon
SWT (S)	Ku_c12447_2002	2B	703976259	4.28-4.68	exon
SWT (S)	IACX5941	2B	712600539	2.99-3.26	exon
SWT (S)	Excalibur_c80601_278	2B	713676010	3.95-4.32	exon
SWT (S)	Excalibur_c27629_473	2B	714132936	3.87-4.22	exon
SWT (S)	IAAV3100	2B	715028611	3.23-3.48	exon
SWT (S)	wsnp_Ex_c28627_37743031	2B	715029293	3.17-3.41	exon
SWT (S)	wsnp_Ex_c24135_33382521	2B	715030098	3.23-3.48	exon
SWT (S)	Ra_c13298_783	2B	732344504	3.24-3.58	exon
SWT (S)	BS00022805_51	2B	745719282	3.12-3.27	exon

GWAS using MLM model identified three SNPs, including BS00021943_51 associated with RLT, Kukri_c12563_52 and wsnp_Ex_c12812_20324622 associated with SLT under both control and stress conditions. It also detected four pleiotropic SNPs including (i) BS00021943_51 associated with RLT (C), RLT (S), and SLT (C), (ii) BS00022072_51 associated with RLT (S) and SLT (C), (iii) Excalibur_c46878_419 associated with RWT (C) and SWT (C), and (iv) wsnp_Ex_c12812_20324622 associated with RLT (C), SLT (C), and SLT (S) (**Table 5**). Similarly, GWAS using FarmCPU and BLINK models identified four SNPs, including BS00021943_51and BS00022072_51 associated with RLT, Kukri_c12563_52 and wsnp_Ex_c12812_20324622 associated with SLT under both control and stress conditions (**Supplementary Table 2**). It also detected six pleiotropic SNPs located on chromosomes 3B, 4A, 4B, and 7A, which involved the following- BS00021943_51 and BS00022072_51 associated with RLT (C), RLT (S), SLT (C), Excalibur_c46878_419 associated with RWT (C) and SWT (C), Kukri_c19696_60 associated with RLT (C) and RWT (C), RAC875_c1817_950 associated with RLT (S) and SLT (S), and wsnp_Ex_c12812_20324622 associated with SLT (C), SLT (S), and RLT (C) (**Supplementary Table 2**).

**Table 5:** Details on pleiotropic MTAs identified in hydroponics experiment (detected with all three models).

Trait	Significant SNP	Chromosome	Position	-log10(P)
RLT(C), RLT(S), SLT(C)	BS00021943_51	3B	69352897	3.08-4.23
RLT(S), SLT(C)	BS00022072_51	3B	69359638	3.29-3.84
RWT(C), SWT(C)	Excalibur_c46878_419	4B	1782490	3.03-3.59
RLT(C), SLT(S), SLT(C)	wsnp_Ex_c12812_20324622	4A	605731006	3.05-3.94

### Marker trait associations identified under field conditions

GWAS using phenotypic data collected from the field experiments conducted at three different locations during 2021-22 identified several significant MTAs based on the exploratory threshold of -log10(P) = 3.0. Among these, 585 MTAs (146 MTAs with MLM, 209 with FarmCPU, and 230 with BLINK) were detected using BHU field data (**Supplementary Table 3**), 879 MTAs (286 MTAs with MLM, 318 with FarmCPU, and 275 with BLINK) using BISA field data (**Supplementary Table 4**), and 1075 MTAs (283 MTAs with MLM, 418 with FarmCPU, and 374 with BLINK) using the ARI field data (**Supplementary Table 5** and **Supplementary Figures 4A–F**). Among these MTAs, 451 MTAs were identified at different locations using all three models; trait-wise distribution of these MTAs is as follows- 48 for CTDGF(C), 20 for CTDGF(S), 15 for CTDH(C), 5 for CTDH(S), 7 for CTDM(C), 17 for CTDM(S), 35 for DH(C), 5 for DH(S), 24 for DM(C), 40 for DM(S), 37 for GY(C), 24 for GY(S), 52 for NDVIGF(C), 20 for NDVIGF(S), 13 for NDVIH(C), 7 for NDVIH(S), 19 for NDVIM(C), 8 for NDVIM(S), 34 for PH(C), 32 for PH(S), 21 for TKW(C), and 7 for TKW(S). Among these, 168 MTAs were associated with different traits recorded under stress conditions (**Supplementary Table 6**). Fourteen of the 451 MTAs were found to be associated with the same traits under both control and stress conditions (**Supplementary Tables 3**-**5**). For instance, Excalibur_c34189_122, wsnp_Ex_c6142_10746442, and wsnp_Ra_c2063_4012957 (all located on 7A) were associated with GY under both optimum and stress conditions, whereas BobWhite_c5654_231, BS00110124_51, BS00110642_51 (all located on 7D) were associated with PHT under both optimum and stress conditions at different locations (**Supplementary Tables 3**-**5**).

Furthermore, the 451 MTAs detected using all three models also included as many as 29 MTAs, each associated with at least two traits, either at the same or different locations (**Table 6**). These 29 MTAs were located on chromosomes 1B, 1D, 2D, 3A, 3D, 4B, 4D, 5A, 6B, 7A and 7B. For instance, BS00022299_51 (5A) was associated with NDVIGF(S), NDVIM(S), NDVIGF(C), and NDVIM(C); Excalibur_c1205_188 (7B) was associated with DH(C), DM(C), and CTDH(C); Excalibur_rep_c106790_155 (4D) was associated with DH(C), PH (S), and DM(C); IAAV4799 (5A) was associated with NDVIM(S), NDVIM(C), CTDGF(S), and CTDM(S); wsnp_Ex_c12223_19533198 (2D) was associated with NDVIH(C), NDVIGF(C), NDVIM(C), and NDVIH(S) and wsnp_Ex_c12850_20377830 (3A) was associated with PH(C), GY(C), and DM(S).

**Table 6:** Details on pleiotropic MTAs identified in field experiments (detected with all 3 models).

Trait	Location	Significant SNP	Chromosome	Position	-log10(P)
NDVIGF (C), NDVIM(C)	Pune	BobWhite_c28409_271	6B	635175572	3.25-4.32
NDVIGF (C), NDVIM(C)	Pune	BobWhite_c3194_125	6B	637024384	3.11-4.03
DM(C), CTDM(C)	Jabalpur	BobWhite_c34030_310	4B	547332570	3.01-3.45
NDVIGF(S), NDVIM(S), NDVIGF(C), NDVIM(C)	Jabalpur	BS00022299_51	5A	679740028	3.37-7.79
NDVIGF(S), NDVIH(S)	Jabalpur	BS00067117_51	3D	596675272	3.03-3.13
NDVIH(C), NDVIM(C)	Jabalpur	BS00071424_51	7A	636898819	3.79-9.77
NDVIGF(C), TKW(C)	Jabalpur, Pune	BS00093325_51	1D	2219936	3.63-5.52
DH(C), DM(C), CTDH(C)	Jabalpur, BHU	Excalibur_c1205_188	7B	709522057	3.00-3.24
NDVIGF(C), NDVIM(C)	Pune	Excalibur_c17905_126	6B	639148172	3.11-4.03
NDVIGF(C), NDVIM(C)	Pune	Excalibur_c6260_536	6B	420958180	3.47-4.69
DH(C), PH (S), DM(C)	Jabalpur	Excalibur_rep_c106790_155	4D	113155391	3.13-3.62
NDVIGF(C), NDVIM(C)	Pune	GENE-3807_45	6B	634331938	3.27-4.50
NDVIM(S), NDVIM(C), CTDGF(S), CTDM(S)	BHU, Pune	IAAV4799	5A	598666287	3.02-3.78
NDVIGF(C), NDVIM(C)	Pune	Kukri_c1836_1167	6B	420958866	3.06-4.31
NDVIGF(C), NDVIM(C)	Pune	Kukri_c25082_328	6B	635172419	3.11-4.02
NDVIGF(C), NDVIM(C)	Pune	Kukri_c6128_373	6B	634333618	3.27-4.50
NDVIGF(C), NDVIM(C)	Pune	Kukri_rep_c104521_117	6B	634333465	3.27-4.50
NDVIGF(C), NDVIM(C)	Pune	Kukri_rep_c104521_601	6B	634332420	3.27-4.50
NDVIGF(C), NDVIM(C)	Pune	Kukri_rep_c104521_727	6B	420958929	3.47-4.69
DH(C), NDVIGF(S)	Jabalpur	Ra_c9938_1299	5A	486750039	3.24-4.41
DH(C), NDVIGF(S)	Jabalpur	Ra_c9938_1734	5A	486750616	3.24-4.41
DH(C), DM(C)	Jabalpur	RAC875_c10372_671	7B	532167768	3.16-3.49
NDVIGF(C), NDVIM(C)	Pune	RAC875_c1349_270	6B	634334367	3.27-4.50
NDVIGF(C), NDVIM(C)	Pune	RAC875_c7965_80	6B	634333384	3.33-4.55
NDVIH(C), NDVIM(C)	Jabalpur	Tdurum_contig94450_255	1B	671199303	3.85-9.17
NDVIH(C), NDVIGF(C), NDVIM(C), NDVIH(S)	BHU	wsnp_Ex_c12223_19533198	2D	742814954	3.02-4.42
PH(C), GY(C), DM(S)	Pune, Jabalpur	wsnp_Ex_c12850_20377830	3A	507766112	3.06-3.94
NDVIH(S), NDVIM(C)	Pune and Jabalpur	wsnp_Ex_c20250_29303152	3A	696243396	3.58-3.74
GY(C), PH(C)	Pune and Jabalpur	wsnp_JG_c2509_1153697	3A	131458607	3.02-3.56

### Evaluation of allelic effects for some selected SNPs

Among the MTAs available for various seedling traits recorded under stress conditions, two highly significant MTAs (detected with all three models) for each of the following traits-RLT (BS00021943_51 and RAC875_c1817_950), RWT (RAC875_rep_c107228_92 and Tdurum_contig60421_74), SLT (Kukri_c12563_52 and wsnp_Ex_c12812_20324622), and SWT (Excalibur_c80601_278 and Ku_c12447_2002), and one highly significant MTA (Tdurum_contig49841_618) associated with CL (detected with two models) were selected to evaluate the effects of their alleles on respective traits under stress conditions (**Supplementary Table 7**). The results revealed significant differences between the allelic effects for all the studied traits. For instance, significant differences (*P*= *2.72e-04*) between the alleles of Tdurum_contig49841_618 associated with CL were observed, where favorable allele improved average CL by the value of 4.94 (**Supplementary Figure 5A**). Similarly, significant differences (*P*= *4.15e-04*) between the alleles of BS00021943_51 associated with RLT were observed, where favorable allele improved average RLT by 2.71 cm (**Supplementary Figure 5B**). In the same way, significant differences (*P*= *7.98e-04* and *6.26e-04*) between the alleles of RAC875_rep_c107228_92 and Tdurum_contig60421_74 associated with RWT were observed, where favorable alleles improved average RWT by the values of 0.06 and 0.05 (g), respectively (**Supplementary Figures 5C, D**).

Similarly, among the MTAs available for some traits recorded under stress conditions in different field experiments, some stable and significant SNPs including wsnp_Ex_c48789_53586502, Tdurum_contig42858_1256, Tdurum_contig42858_1352, wsnp_Ex_c48789_53586406, RAC875_c29455_79, tplb0035a03_368, RAC875_c8721_212, and Ra_c11721_631 associated with DM(S); Kukri_c6274_1283, BS00009565_51, wsnp_Ex_c9618_15912364, and RAC875_c5834_235 associated with PH(S); Excalibur_c3286_103, wsnp_Ex_c6142_10746442, wsnp_Ra_c7112_12318340, wsnp_Ex_c53387_56641291, BobWhite_c25527_313, wsnp_Ra_c2063_4012957, and Excalibur_c34189_122 associated with GY(S) were selected to evaluate the effects of their alleles on respective traits (combined BLUEs) under stress conditions (**Supplementary Table 7**). The analysis revealed significant differences between the allelic effects of different SNPs for all the studied traits. For instance, significant differences (*P*= *2.08E-03*) between the alleles of RAC875_c8721_212 associated with DM(S) were observed, where one allele reduced the average DM(S) by 2.53 days (**Supplementary Figure 6A**). Similarly, significant differences (6.45E-03 and 8.41E-03) were reported between the alleles of Kukri_c6274_1283 and BS00009565_51 associated with PH(S), with favorable alleles reducing the average PH(S) by 2.32 and 2.49 cm, respectively. In the same way, significant differences (with *P value* ranging from *2.69E-04* to *7.17E-04*) between the alleles of Excalibur_c3286_103, wsnp_Ex_c6142_10746442, wsnp_Ra_c7112_12318340 (**Supplementary Figures 6B–D**), wsnp_Ex_c53387_56641291, BobWhite_c25527_313, wsnp_Ra_c2063_4012957, and Excalibur_c34189_122 (**Supplementary Figures 7A–D**) associated with GY(S) were observed, where favorable alleles improved average GY(S) by the values ranging from 41.83 to 54.56 g/plot.

### Haplotypes associated with different seedling traits

**Figure 4:**
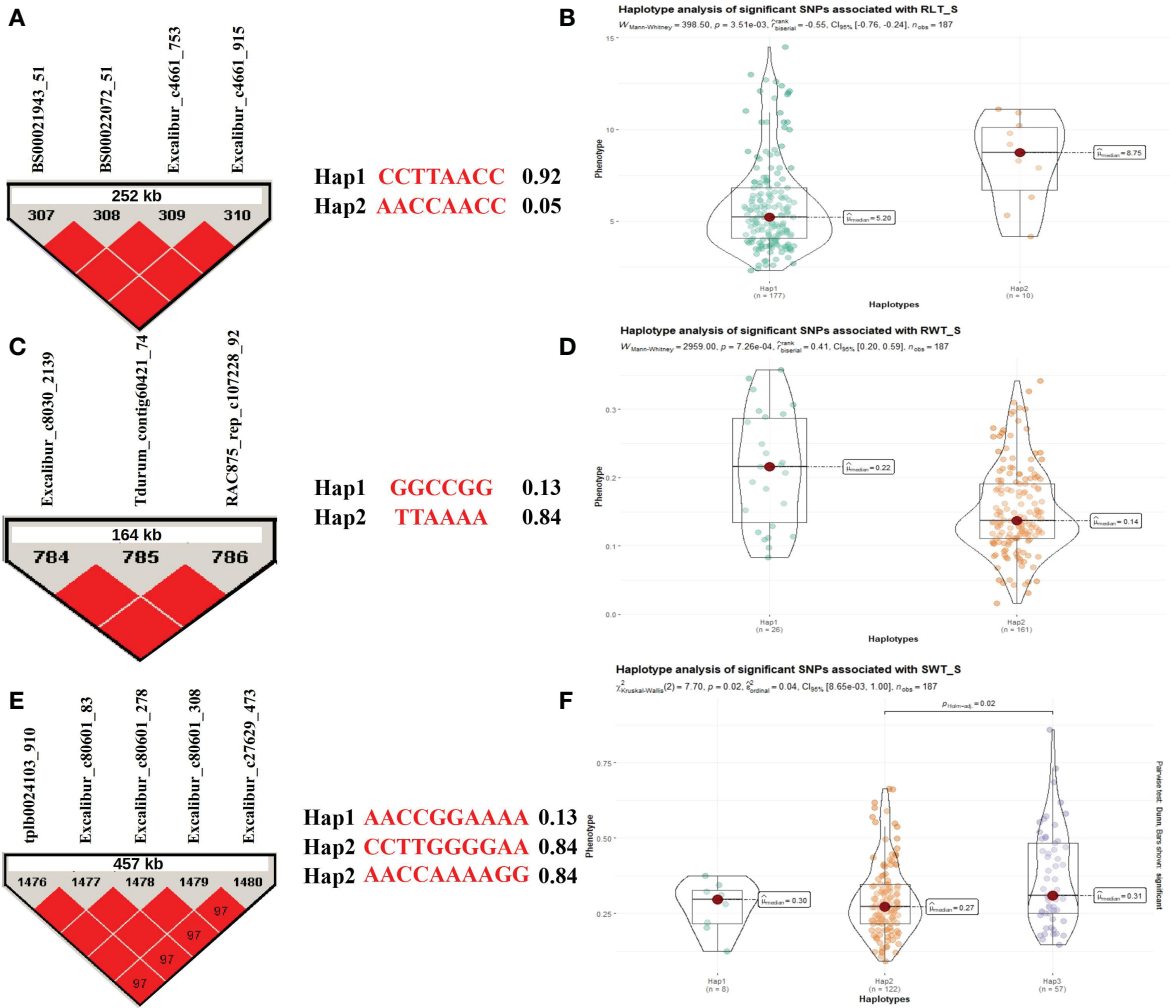
Haplotype analysis for traits recorded under stress conditions during the hydroponics experiment, **(A)** LD block for the 252 kb region harboring MTA for RLT and two allelic haplotypes identified in the panel based on four SNPs present in the LD block along with frequencies for each haplotype; **(B)** Differences in RLT between two haplotypes; **(C)** LD block for the 164 kb region harboring MTA for RWT and two allelic haplotypes identified in the panel based on three SNPs present in the LD block along with frequencies for each haplotype; **(D)** Differences in RWT between two haplotypes; **(E)** LD block for the 457 kb region harboring MTA for SWT and three allelic haplotypes identified in the panel based on five SNPs present in the LD block along with frequencies for each haplotype; **(F)** Differences in SWT among three haplotypes.

Some MTAs (as selected above; **Supplementary Table 7**) associated with different seedling traits (1 MTA for CL and 2 MTAs for RLT, RWT, SLT, and SWT each) under stress condition were utilized for the haplotype analysis. Based on the allelic distribution of the SNPs in the WAMI panel, for RLT under hydroponic stress conditions, we identified three haplotypes (*Hap1*, *Hap2*, and *Hap3*; involving a total of 4 SNPs) with a frequency of 0.92, 0.05, and 0.02, respectively. Only first two haplotypes were compared for variations in trait means because *Hap3* had a very low frequency. The analysis revealed significant differences (*P* = *6.26e–04*) between two haplotypes (*Hap1* and *Hap2*) for RLT, where *Hap2* (8.31g) had higher average RLT compared to *Hap1*(5.83g) (**Figures 4A, B**). Similarly, we identified three haplotypes (*Hap1*, *Hap2*, and *Hap3*; involving a total of 3 SNPs) for RWT with a frequency of 0.13, 0.84, and 0.01, respectively. Similarly, only first two haplotypes were compared for variations in trait means because *Hap3* had a very low frequency. The analysis revealed significant differences *(P* = *7.26e–04*) between two haplotypes (*Hap1* and *Hap2*) for RWT, where *Hap1* (0.21g) had a higher average RWT compared to *Hap2*(0.15g) (**Figures 4C, D**). In the same way, we identified three haplotypes (*Hap1*, *Hap2*, and *Hap3*; involving a total of 5 SNPs) for SWT with a frequency of 0.04, 0.63, and 0.29, respectively. The analysis revealed significant differences (*P* = *8.65e-03*) between the *Hap2* and *Hap3* for SWT, where *Hap2* (0.36g) had higher average SWT compared to *Hap3*(0.29g) (**Figures 4E, F**). There was no significant variation among the SLT haplotypes for the trait means, whereas, no haplotype was detected for the SNPs associated with CL.

**Figure 5:**
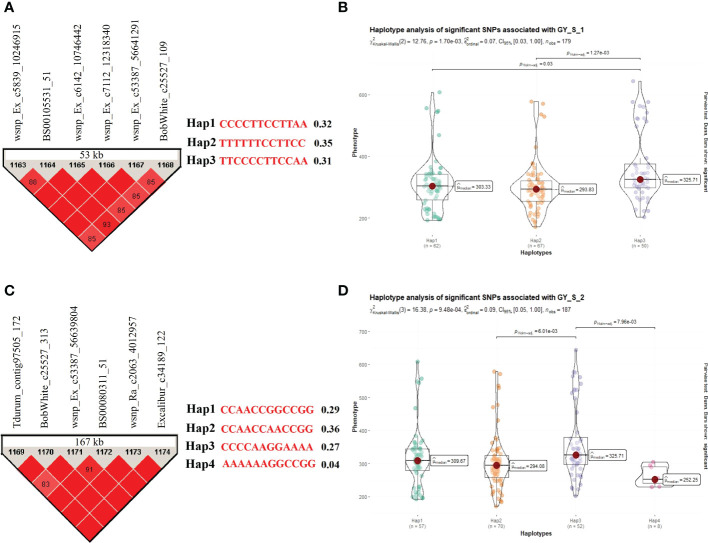
Haplotype analysis for traits recorded under stress conditions during the field experiments, **(A)** LD block for the 53 kb region harboring MTA for GY and three allelic haplotypes identified in the panel based on six SNPs present in the LD block along with frequencies for each haplotype; **(B)** Differences in GY among three haplotypes; **(C)** LD block for the 167 kb region harboring MTA for GY and four allelic haplotypes identified in the panel based on six SNPs present in the LD block along with frequencies for each haplotype; **(D)** Differences in GY among four haplotypes.

Similarly, seven stable and significant SNPs (Excalibur_c3286_103, wsnp_Ex_c6142_10746442, wsnp_Ra_c7112_12318340, wsnp_Ex_c53387_56641291, BobWhite_c25527_313, wsnp_Ra_c2063_4012957, and Excalibur_c34189_122) on chromosome 7A associated with GY under stress condition reported in the field experiments were utilized for the haplotype analysis. Based on the allelic distribution of these SNPs in the WAMI panel, we identified two sets of haplotypes for GY under stress conditions in field experiments. In the first group, we identified six haplotypes (*Hap1*, *Hap2*, *Hap3*, *Hap4*, *Hap5*, and *Hap6*; involving a total of six SNPs) with a frequency of 0.32, 0.35, 0.31, 0.02, 0.01, and 0.01, respectively. Only first three haplotypes were compared for variations in trait means because the last three haplotypes (viz., *Hap4*, *Hap5*, and *Hap6*) had very low frequencies (**Figures 5A, B**). The analysis revealed significant differences between haplotypes *Hap1* and *Hap3* (*P*=*0.03*) and between haplotypes *Hap2* and *Hap3* (*P*=*1.27E-03*) for GY(S). Among these three haplotypes, *Hap3* (mean = 361.16 g/plot) had maximum GY followed by *Hap1* (mean =310.47 g/plot) and *Hap2* (mean GY(S) =300.42 g/plot). In the second group, we identified five haplotypes (*Hap1*, *Hap2*, *Hap3*, *Hap4*, and *Hap5*; involving a total of six SNPs) with a frequency of 0.29, 0.36, 0.27, 0.04, and 0.005, respectively. Among these five haplotypes, first four haplotypes were compared for variations in trait means because the last haplotype (viz., *Hap5*) had a very low frequency (**Figures 5C, D**). The analysis revealed significant differences between haplotypes *Hap2* and *Hap3* (*P*=*6.01E-03*) and between haplotypes *Hap3* and *Hap4* (*P*=*7.96E-03*) for GY(S). Among these four haplotypes, *Hap3* (mean = 360.75 g/plot) had maximum GY followed by *Hap1* (mean =317.49 g/plot), *Hap2* (mean =305.29 g/plot), and *Hap4* (mean =262.16 g/plot).

### Candidate genes and expression analysis

Gene annotation and *in silico* expression analysis revealed as many as 102 non-redundant important candidate genes (CGs) underlying the nine stable genomic regions (as selected above) detected under stress conditions in hydroponic conditions. These genes encode several important proteins such as F-box-like protein, ABC transporter-like protein, GDSL lipase/esterase, glycosyltransferase, AP2/ERF TF, protein kinase, calcineurin-like phosphodiesterase, GroES-like superfamily protein, WD40 repeat and others (**Supplementary Table 8**). The above candidate genes were also subjected to an *in* silico expression analysis utilizing RNA-seq data available from a public database. As many as 23 CGs from different MTAs exhibited differential expression, and these differentially expressed CGs (DECGs) were divided into up-regulating (FC ≥ 2) and down-regulating (FC ≤ − 2) CGs (**Supplementary Figure 8** and **Supplementary Table 10**). These 23 genes primarily encoded for the following proteins: protein kinases, GroES-like superfamily proteins, OTU domain containing proteins, KIX domain superfamily proteins, NAD-dependent epimerase/dehydratase, etc. A few genes (e.g., *TraesCS2B02G519900* and *TraesCS5B02G035700*) with unknown protein products also showed significant differential expression under control and stress conditions. A representative heat map of these DECGs is represented in **Supplementary Figure 8**.

Similarly, gene annotation and *in silico* expression analysis revealed as many as 43 non-redundant important candidate genes underlying the 10 stable genomic regions including RAC875_c8721_212 (6A), Kukri_c6274_1283 (4A), BS00009565_51 (4A), and Excalibur_c3286_103, wsnp_Ex_c6142_10746442, wsnp_Ra_c7112_12318340, wsnp_Ex_c53387_56641291, BobWhite_c25527_313, wsnp_Ra_c2063_4012957, and Excalibur_c34189_122 (each located on 7A and associated with GY) detected under stress conditions in field experiments (**Supplementary Table 9**). The above CGs were also subjected to an *in* silico expression analysis utilizing RNA-seq data available from a public database. Up to seven CGs exhibited differential expression, and these DECGs were divided into up-regulating (FC ≥ 2) and down-regulating (FC ≤ − 2) CGs (**Supplementary Figure 8** and **Supplementary Table 10**). These genes are known to encode several important proteins such as protein kinases, Bromodomain, Armadillo, Cyanobacterial aminoacyl-tRNA synthetase, O-methyltransferase domain containing proteins, etc.

## Discussion

Breeding for drought tolerance deserves much higher attention and investment to sustain wheat production under the current climate change scenario. Wheat is affected by drought stress at all growth stages. Even though most earlier studies focused on aboveground adult plant traits (El Gataa et al., 2022), seedling traits are equally important since the seedling stage is the most vulnerable stage in warm and dry regions, and their trait values may differ significantly from those of adult plants (Harrison and Laforgia, 2019). This study evaluated a bread wheat panel systematically assembled from the CIMMYT gene bank for seedling traits (viz., CL, SLT, RLT, SWT, and RWT) under hydroponics conditions. The interaction between water treatments and genotypes was highly significant for all the traits studied, indicating substantial genotypic variation in response to water treatments. Significant differences between water treatments and wheat genotypes have been reported in earlier studies (e.g., El Gataa et al., 2022; Sallam et al., 2022).

All the traits evaluated during hydroponics and field experiments reduced significantly but variably under drought stress, indicating the variation in the phenotypic plasticity of the studied traits. Phenotypic plasticity is one of the major mechanisms of adaptation to abiotic stresses through modifications in critical developmental stages (Fatiukha et al., 2021). Various genotypes that exhibited drought tolerance either at the seedling or adult plant stages could be deployed in the breeding programs to achieve tolerance at the desired stage. A few genotypes that demonstrated drought tolerance at both stages appear to be a better choice for use as parents in the breeding program targeting drought stress tolerance. We observed moderate to high heritability estimates for different traits in the hydroponics experiment as obtained in some of the earlier studies conducted under control and water stress conditions (Maulana et al., 2020; Danakumara et al., 2021). Moderate to high broad-sense heritability for most of the traits implies that it may be useful to employ these traits for a better comprehension of the genetics underlying the yield potential of wheat under drought stress conditions.

GWAS is a popular technique for dissecting the genetic architecture of complex traits in wheat (Sidhu et al., 2020; AlTameemi et al., 2021; Saini et al., 2022; Zhang et al., 2022) and other crops, but this strategy is prone to the identification of false positives owing to confounding population structure, cryptic relatedness of the individuals in the population or the effect of phenology traits. In this study, we used one single locus model (i.e., MLM) and two multi-locus models (viz., FarmCPU and BLINK), which have been known to provide the most reliable results (reviewed in Saini et al., 2022). Further, we used the genotypes with similar heading dates for GWAS therefore, minimizing the effect of phenology on the GWAS results. In addition, the Infinium iSelect 90K assay is known to detect polymorphism in allo-hexaploid wheat populations by analyzing over 81,000 gene-associated SNPs (Wang et al., 2014). This SNP array is known for providing greater genome coverage and resolution during the dissection of different complex traits in wheat (Muhu-Din Zhu et al., 2019; Ahmed et al., 2020; Muhammad et al., 2020; Zhang et al., 2021). Unlike several previous studies (Ahmed et al., 2020; Muhammad et al., 2020; Zhang et al., 2021), we considered the physical positions of the SNPs for GWAS analysis, which is consistent with a few previous studies (Zhu et al., 2019; Safdar et al., 2020) using the 90K Infinium iSelect assay for GWAS.

Further, it is essential to establish the *p*-value threshold. A threshold that is too lenient will flag a false-positive association (a type I error), while one that is too strict will likely miss a true association (a type II error). In this study, we employed an exploratory threshold [-log10(P) = 3.00] based on independent tests to consider any SNP as significant in individual environments, as reported in some previous studies (Halder et al., 2019; Kumar et al., 2021; Gill H. S. et al., 2022).

Most previous studies have identified the genomic regions associated to drought stress tolerance in wheat either at seedling (Maulana et al., 2020; Danakumara et al., 2021; Sallam et al., 2022) or at the reproductive stage (Gataa et al., 2021; El Gataa et al., 2022). However, a few studies conducted GWAS for traits on both seedlings and adult plant stages (e.g., Rufo et al., 2020). The WAMI panel used in this study has previously been studied to screen for drought tolerance under rainfed conditions (Gahlaut et al., 2019; Gahlaut et al., 2021). However, our study is unique in that we imposed drought treatment specifically at the flowering stage, during which soil moisture levels reached approximately 45%. In addition to the field trials, we also conducted hydroponic experiments to identify genomic regions associated with drought tolerance at the seedling stage.

In this study, 94 MTAs associated with various seedling traits were identified using all three models under either normal or stress or both conditions. Two of these MTAs were previously detected to be associated with different seedling traits on chromosomes 3A and 7D (Maulana et al., 2020). The remaining MTAs identified on chromosomes 1A, 2A, 2B, 3A, 3B, 4A, 4B, 5A, 5B, 5D, 7A, 7B, and 7D have not been previously reported and they can be considered novel. In addition, several significant SNPs were reported to be associated with seedling traits under both normal and stress conditions. For instance, BS00021943_51 and BS00022072_51 (RLT), Kukri_c12563_52 and wsnp_Ex_c12812_20324622 (SLT). Some significant SNPs each associated with at least two different seedling traits were also detected, for instance, BS00021943_51 and BS00022072_51 associated with RLT (C), RLT (S), SLT (C), Kukri_c19696_60 associated with RLT (C) and RWT (C), and wsnp_Ex_c12812_20324622 associated with SLT (C), SLT (S), and RLT (C). Earlier studies have also reported a significant association between root and shoot traits (Figueroa-bustos et al., 2018) and shared genetic control of these traits (Danakumara et al., 2021).

Similarly, 451 MTAs associated with different adult plant traits were identified at different locations under normal or stress conditions. Seventy-one of these MTAs were previously detected to be associated with different adult plant traits recorded under normal and drought stress conditions on all 21 chromosomes except 1D, 4B, 4D, and 6B (Mwadzingeni et al., 2017; Bhatta et al., 2018; Sukumaran et al., 2018; Afzal et al., 2019; Gahlaut et al., 2019; Li et al., 2019; Mathew et al., 2019; Qaseem et al., 2019; Ahmed et al., 2020; Shokat et al., 2020; Abou-Elwafa and Shehzad, 2021; Amalova et al., 2021; Rabbi et al., 2021). The remaining MTAs have not been reported and therefore, can be considered novel. Further, several significant SNPs were reported to be associated with adult plant traits under both normal and stress conditions at different locations. Similar results have also been reported in some earlier studies (Maulana et al., 2020; Danakumara et al., 2021; Gataa et al., 2021; Sallam et al., 2022).

As many as 19 significant SNPs each associated with at least two different adult plant traits were also detected, for instance, BS00022299_51 was reported to be associated with NDVIGF(C&S) and NDVIM(C&S); Excalibur_rep_c106790_155 was reported to be associated with DH(C), PH(S), and DM(C); IAAV4799 was found to be associated with NDVIM(C&S), CTDGF(S), and CTDM(S); wsnp_Ex_c12223_19533198 was reported to be associated with NDVIH(C&S), NDVIGF(C), and NDVIM(C), and wsnp_Ex_c12850_20377830 was found to be associated with PH(C), GY(C), and DM(S). A significant positive correlation between NDVIGF and NDVIM has been reported (Sultana et al., 2014). Additionally, Hassan et al., 2019 described that NDVI measured at the grain filling stage may be a useful tool for predicting wheat yield. Stay-green, calculated as NDVI at physiological maturity showed a positive correlation with yield in heat and heat combined with drought environments (Lopes and Reynolds, 2012). There was a significant positive linear relationship between CTDGF and CTDM, as well as between CTDGF and GY. Further, a cooler plant canopy during mid-grain filling in wheat appeared to be an important indicator of greater drought tolerance and yield under dryland conditions (Thapa et al., 2018).

Overall, there were significant differences in the distribution of MTAs associated with the seedling and adult plant traits on different chromosomes, with 45.50% of MTAs identified on the A sub-genome, 37.79% in the B sub-genome, and only 16.69% in the D sub-genome. These findings align with the previous findings of lower genetic diversity and occurrence of higher LD in the D genome (Mwadzingeni et al., 2017; Bhatta et al., 2018; Sukumaran et al., 2018; Abou-Elwafa and Shehzad, 2021; El Gataa et al., 2022; Rabieyan et al., 2022). Furthermore, among the MTAs detected using all three models, as many as 19 significant SNPs associated with adult plant traits at different locations were found to be overlapped with as many as 13 significant SNPs associated with different seedling traits. Relationships between seedling and adult plant traits under drought stress have also previously been reported (Dodig et al., 2015; Rufo et al., 2020).

As many as 13 significant SNPs identified using the data from field experiments on chromosomes 3A, 4A, 4B, 5A, 6B, 7A, 7B, and 7D were found to be overlapping with several major genes known to be associated with various yield component traits under either optimum or stress conditions; these genes included the following- *TaGS5-3A* (Ma et al., 2016), *TaTGW6-A1* (Hanif et al., 2016), *6-SFT-A2* (Yue et al., 2015), *TaSnRK2.10-4B* (Zhang Z. et al., 2017), *TaSnRK2.9-5A* (Rehman et al., 2019), *TaGL3-5A* (Yang et al., 2019), *TaPRR1-6B* (Sun et al., 2020), *WAPO-A1* (Kuzay et al., 2019), *TaSPL20-7B* (Zhang B. et al., 2017), and *TaGS-D1* (Zhang et al., 2014).These observations indirectly validated the findings of the present study.

Functional annotation and gene expression analysis of genes available from significant genomic regions associated with different seedling traits identified 23 important genes with differential expression under control and stress conditions. These genes encoded a variety of important proteins, which are known to play significant roles during drought stress conditions, such as protein kinases (Lim et al., 2020), GroES-like superfamily proteins (Wang et al., 2004), OTU domain-containing proteins (Zang et al., 2020), KIX domain superfamily proteins (Han et al., 2006), NAD-dependent epimerase/dehydratase (Chaichi et al., 2019), etc. Some genes encoding unpredicted or uncharacterized proteins also showed significant expressions in different plant tissues which may also be utilized for future research. Similarly, functional annotation and gene expression analysis identified seven important genes associated with adult plant traits that had differential expression under control and stress conditions. These genes also encoded a number of important proteins, such as protein kinases (Lim et al., 2020), bromodomain (Duque and Chua, 2003), armadillo (Sharma et al., 2014), cyanobacterial aminoacyl-tRNA synthetase (Shahinnia et al., 2021), O-methyltransferase domain containing proteins (Chun et al., 2021), etc., which are known to play critical roles during drought stress conditions.

## Conclusions

The WAMI association panel used in this study showed significant variations for different traits to drought stress recorded at the seedling stage under hydroponic conditions, including CL, SLT, RLT, SWT, and RWT, as well as traits recorded in field experiments, including DTH, DTM, PHT, NDVIH, NDVIGF, NDVIM, CTDH, CTDGF, CTDM, TKW, and GY. This demonstrates that germplasm selection based on these traits may benefit breeding for drought-tolerant wheat genotypes. Contrasting genotypes identified in this study could be used to develop mapping populations for further genetic dissection of the trait(s), while agronomically superior drought-tolerant genotypes could be used as donors in a breeding program. The construction of breeder-friendly Kompetitive allele-specific PCR (KASP) markers for the significant and stable MTAs identified may facilitate the deployment of these genomic regions through marker-assisted selection in the early stages of the wheat breeding process. In addition, the significant MTAs identified during the current study can be integrated into genomic prediction models to evaluate their potential for selection under drought stress conditions. Desirable haplotypes can be used for haplotype-based breeding in wheat to improve yield stability, potential, and performance under drought stress. The identified important genes may also be validated using functional genomics techniques and CG-based association mapping.

## Data availability statement

The original contributions presented in the study are included in the article/**Supplementary Material**. Further inquiries can be directed to the corresponding author.

## Author contributions

SS conceptualized, designed, and monitored the research, SR and GS performed field and hydroponic experiments, DS performed genomic analysis, SR, DS, and GS prepared the initial draft, and SS, VM and AJ edited and finalized the manuscript. All authors contributed to the article and approved the submitted version.
