# Supplementary material for: Genome-wide association mapping of genomic regions associated with drought stress tolerance at seedling and reproductive stages in bread wheat
Source: Front Plant Sci. 2023 May 12;14:1166439. doi: 10.3389/fpls.2023.1166439 (PMC10213333; doi:10.3389/fpls.2023.1166439)
Supplement: Supplementary file 1 [file DataSheet_1.docx]

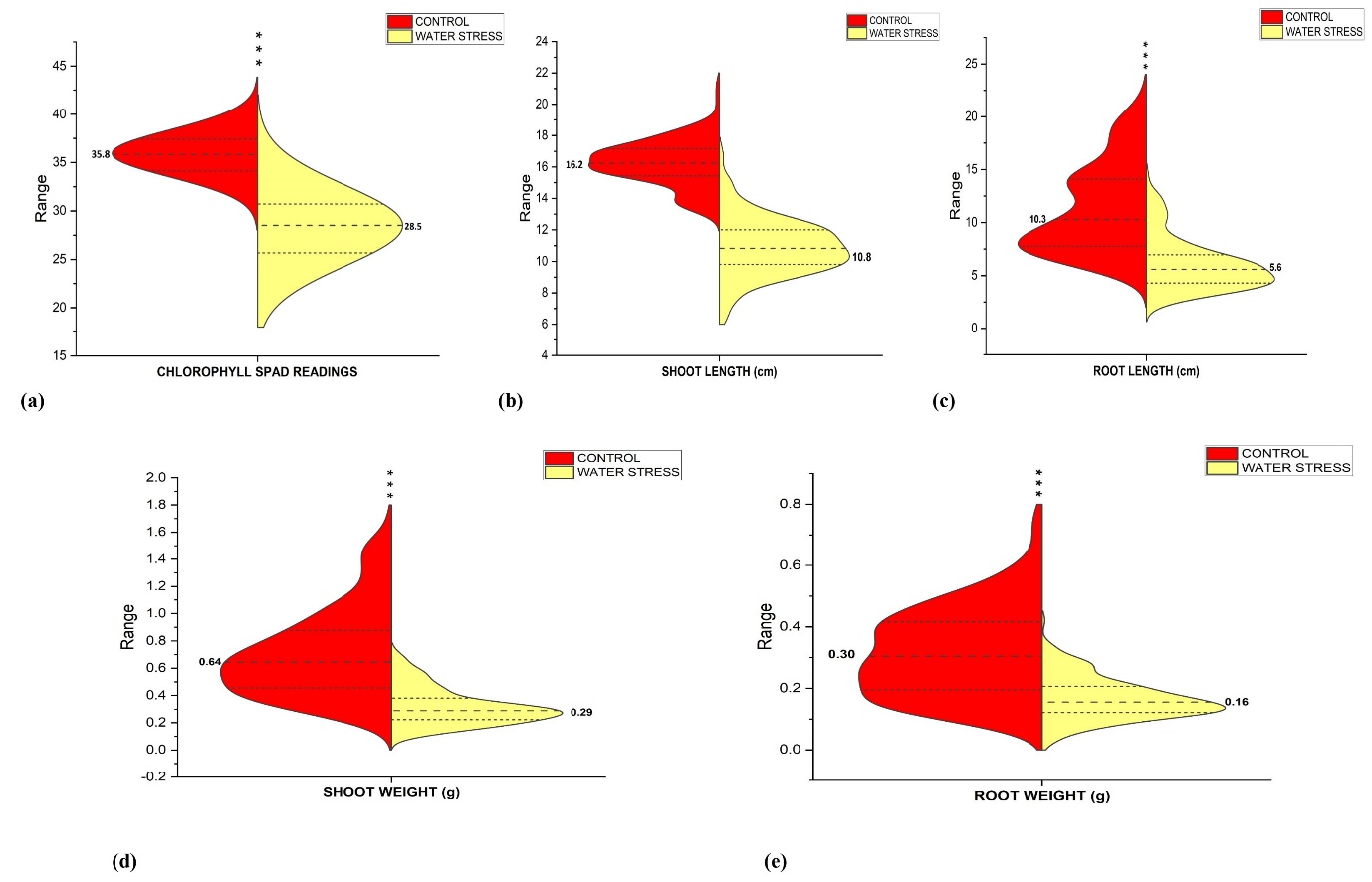


**PD=20.4%**

**PD= 33.3%**

**PD= 45.6%**

**PD= 46.7%**

**PD= 54.7%**

**Supplementary Figure 1:** Distribution of trait values recorded under normal and stress conditions in the hydroponic experiment, (a) chlorophyll content, (b) shoot length, (c) root length, (d) shoot weight, and (e) root weight. PD is the percent decline under water stress compared to control


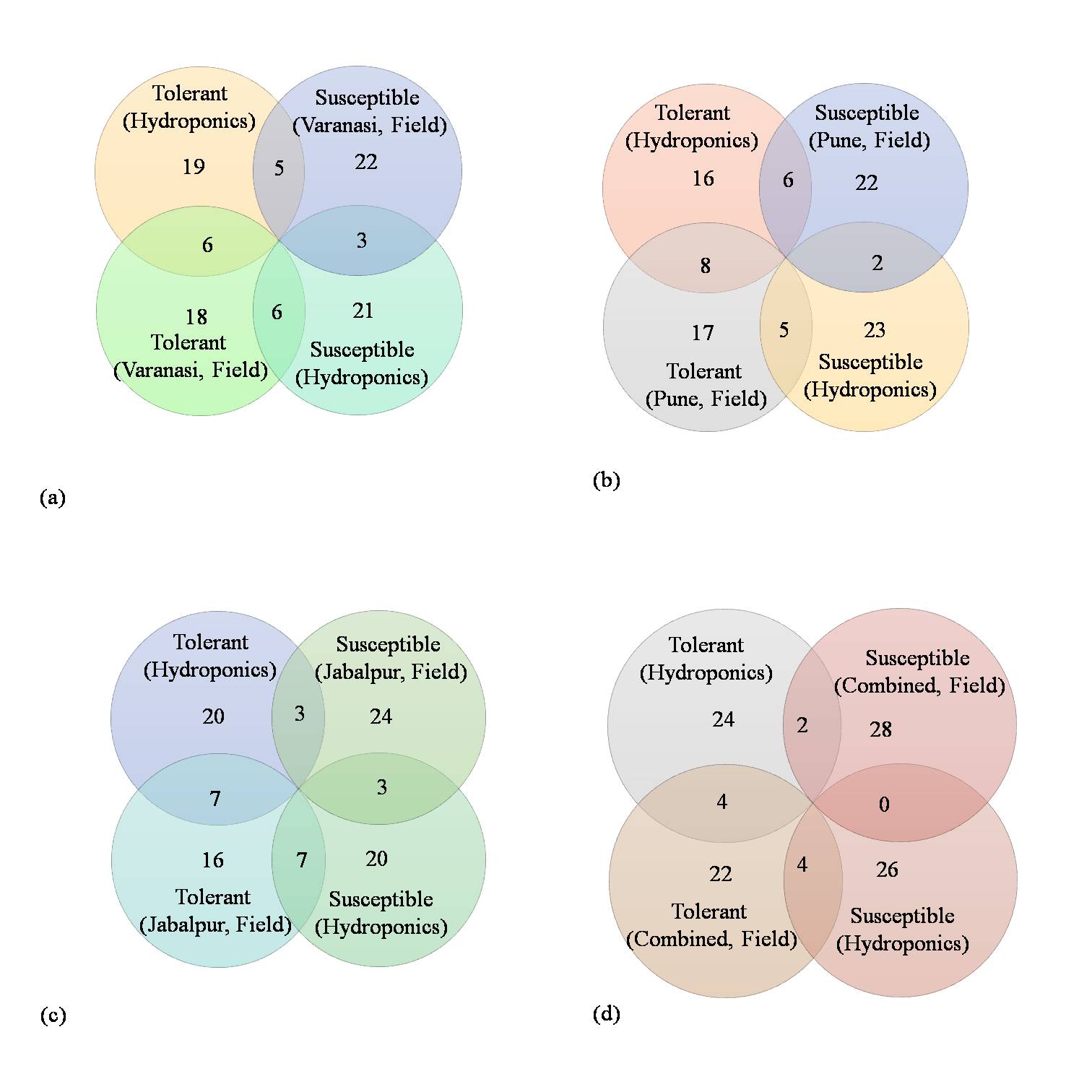


**Supplementary Figure 2:** Venn diagrams showing number of genotypes showing differential responses under stress conditions during hydroponics and field experiments, (a) hydroponics v/s Varanasi field; (b) hydroponics v/s Pune field; (c) hydroponics v/s Jabalpur field; and (d) hydroponics v/s across all the three field experiments (combined)


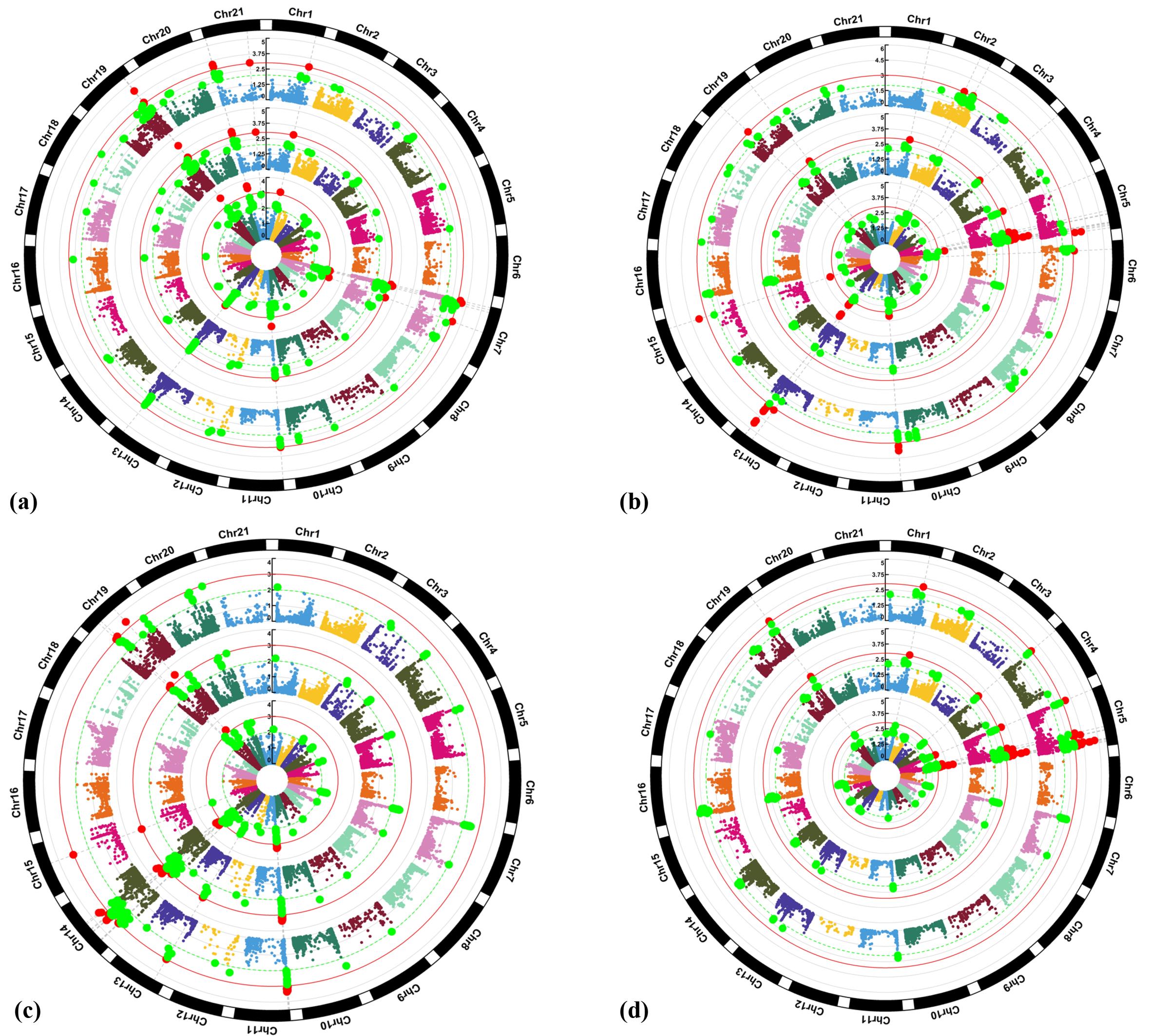


**Supplementary Figure 3:** Manhattan plots of genome-wide association scan using three different models (from inner circle to outer circle: MLM, BLINK, and FarmCPU) for traits phenotyped under control and stress conditions during hydroponics experiment; each dot represents a SNP; the vertical dashed line represents the –log10(P) values. (a) for RWT(C), (b) RWT(S), (c) SWT(C), and (d) SWT(S)


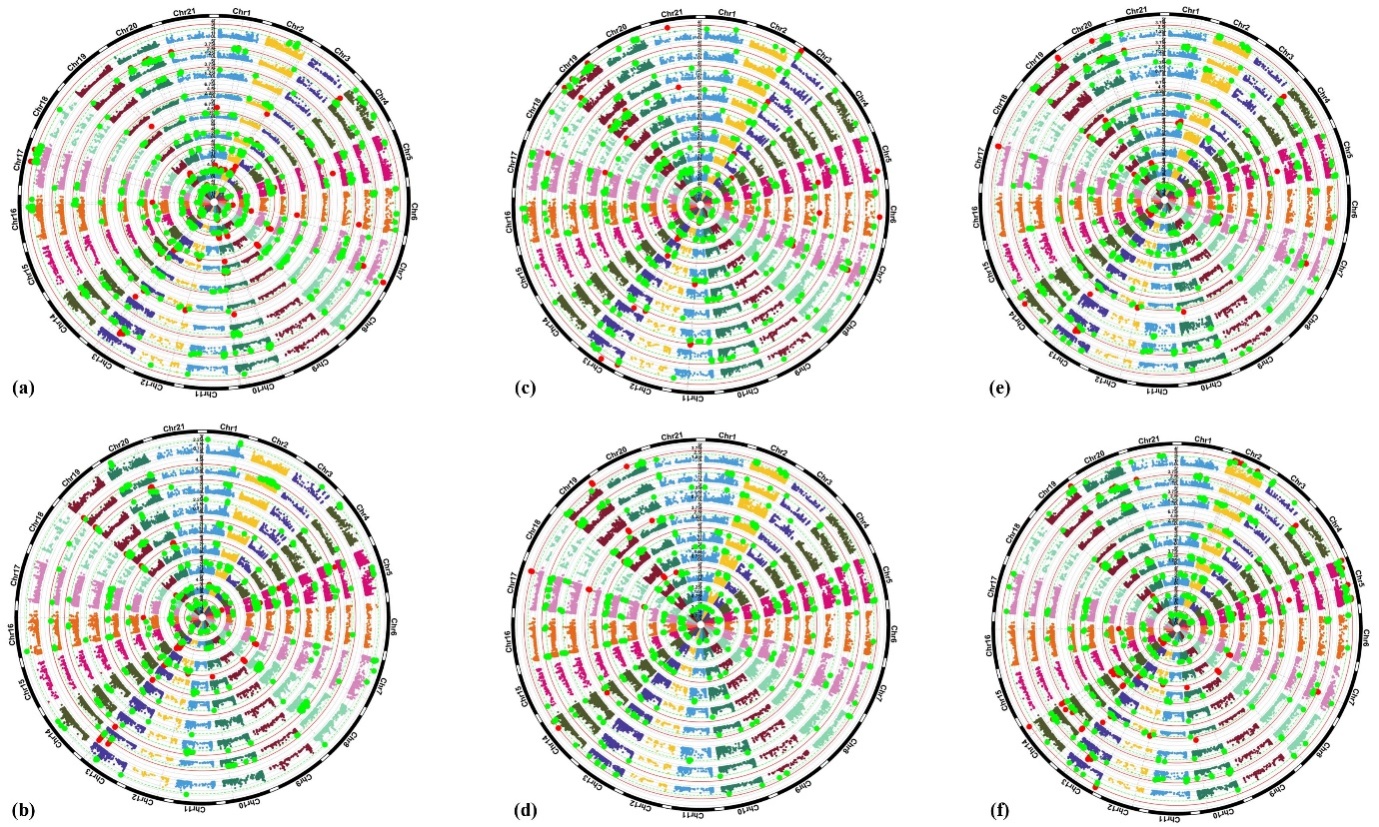


**Supplementary Figure 4:** Manhattan plots of genome-wide association scan using three different models (from inner circle to outer circle: MLM_Varanasi, MLM_Jabalpur, MLM_Pune, BLINK_Varanasi, BLINK_Jabalpur, BLINK_Pune, FarmCPU_Varanasi, FarmCPU_Jabalpur, and FarmCPU_Pune,) for traits phenotyped under control and stress conditions during field experiments; each dot represents a SNP; the vertical dashed line represents the –log10(P) values. (a) for NDVIM(C), (b) NDVIM(S), (c) TKW(C), (d) TKW(S), (e) GY(C), and GY(S)


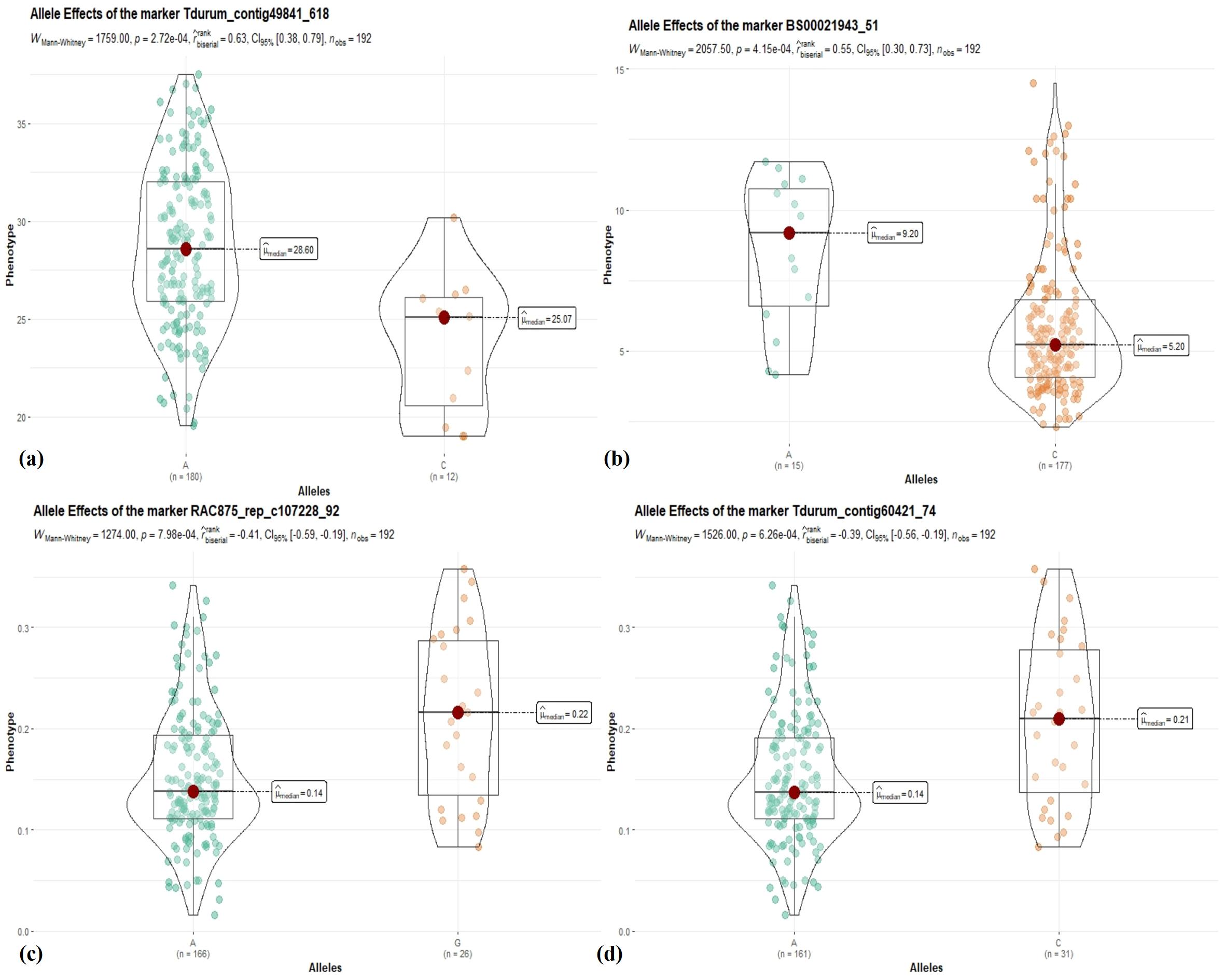


**Supplementary Figure 5:** Showing the effects of two alleles (favorable v/s unfavorable) of the stable MTAs on the trait means for (a) CL, (b) RLT, and (c & d) RWT recorded under stress conditions in hydroponics experiment


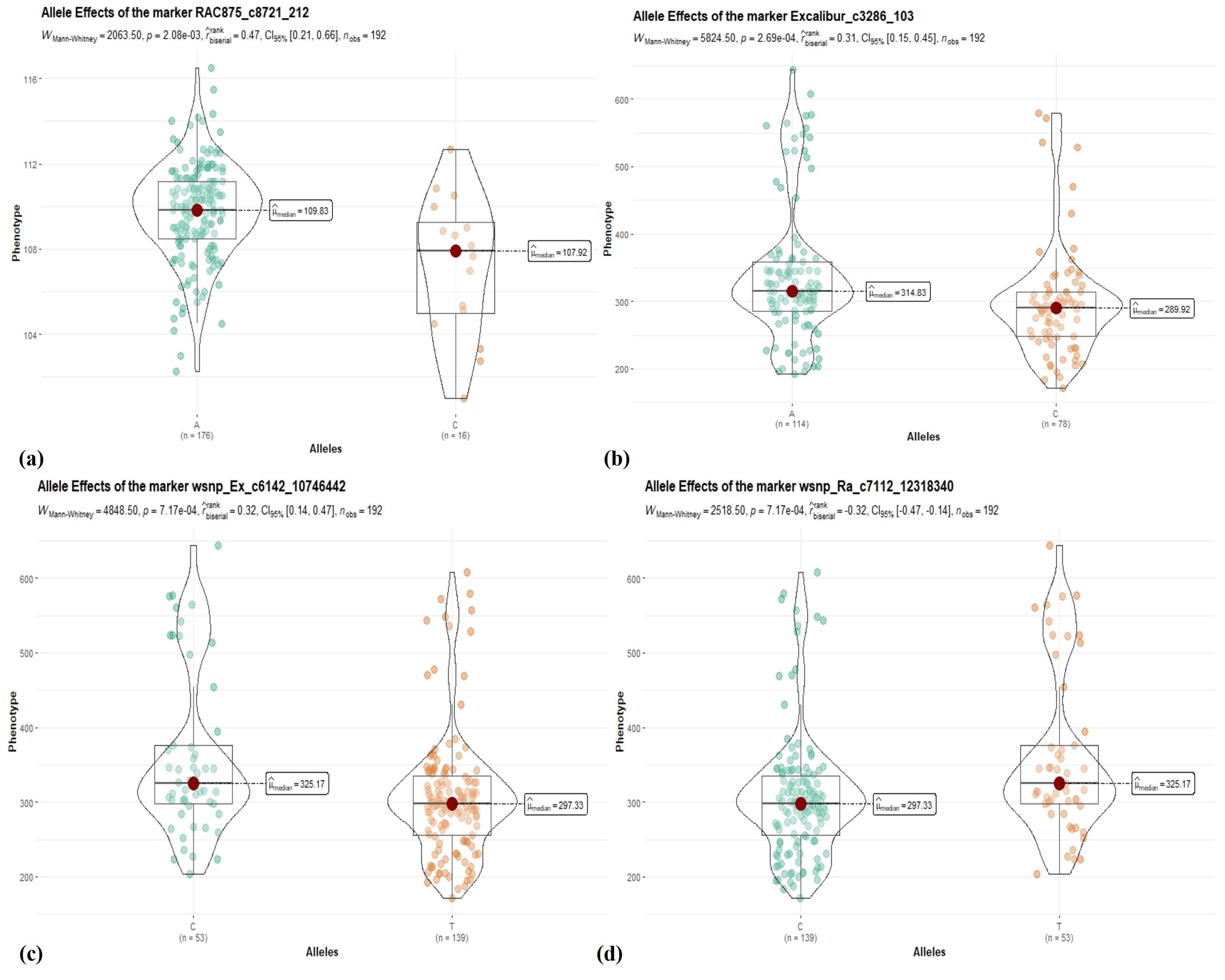


**Supplementary Figure 6:** Showing the effects of two alleles (favorable v/s unfavorable) of the stable MTAs on the trait means for (a) DM, (b, c & d) GY across the three field experiments


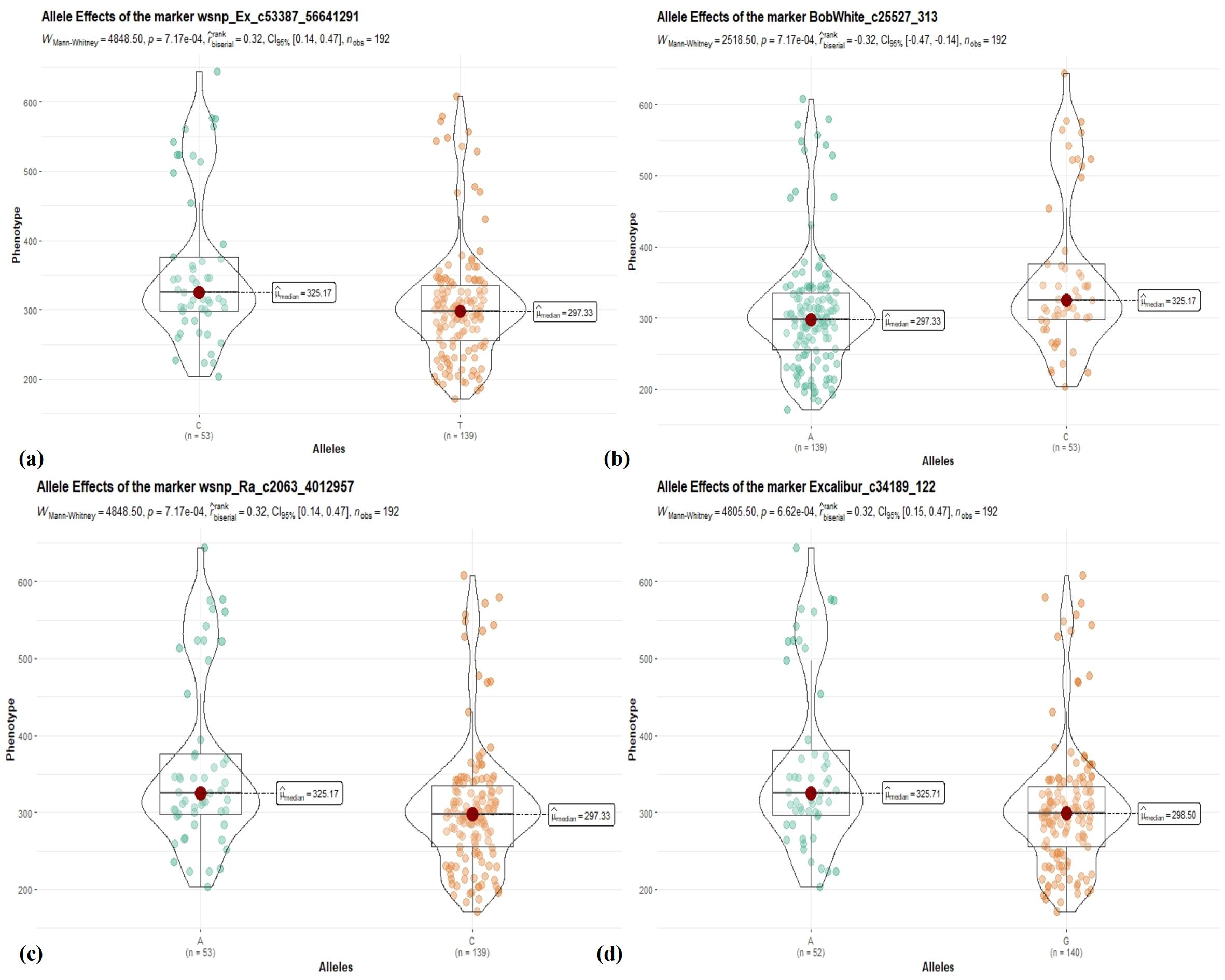


**Supplementary Figure 7:** Showing the effects of two alleles (favorable v/s unfavorable) of the stable MTAs on the trait means for GY across the three field experiments


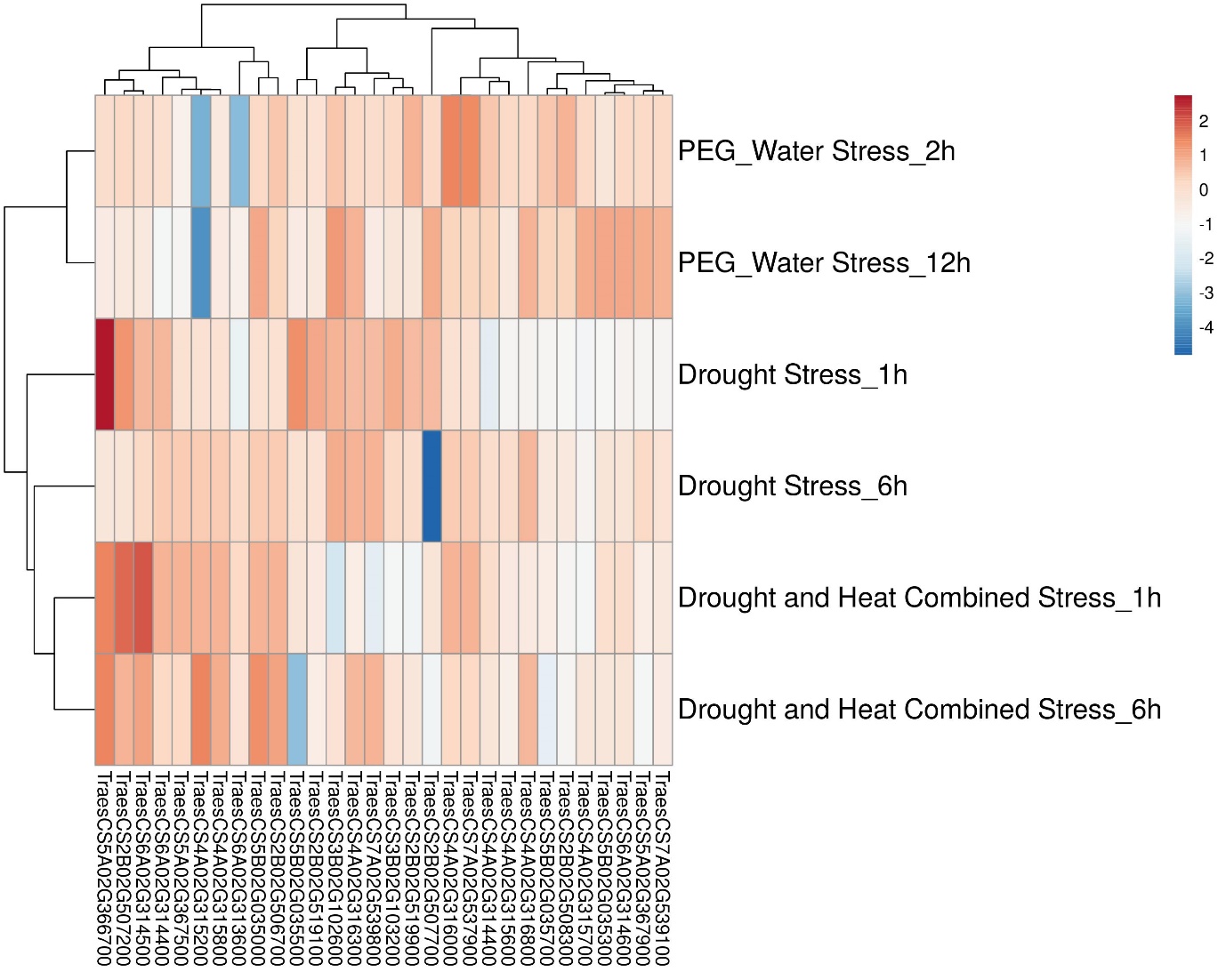


**Supplementary Figure 8:** Heatmaps showing differential expressions (fold changes) of some selected candidate genes under different stress conditions
